# The prognostic value of the systemic immune-inflammation index for patients with bladder cancer after radical cystectomy

**DOI:** 10.3389/fimmu.2022.1072433

**Published:** 2022-11-29

**Authors:** Shiyu Zhang, Jiajia Du, Xin Zhong, Ping Tan, Hang Xu, Jiapeng Zhang, Di Jin, Yifan Li, Weizhen Le, Xingyu Xiong, Tianhai Lin, Qiang Wei

**Affiliations:** ^1^ Department of Urology, Institute of Urology, West China Hospital of Sichuan University, Chengdu, Sichuan, China; ^2^ State Key Laboratory of Biotherapy and Cancer Center, West China Hospital, Sichuan University, Chengdu, Sichuan, China

**Keywords:** systemic immune-inflammation index (SII), bladder cancer, biomarkers, prognosis, radical cystectomy (RC)

## Abstract

**Background:**

Biomarkers acquired from blood samples are easy to obtain and cost-effective, have attracted considerable interest, and have been widely investigated. Inflammation plays a crucial role in cancer cell initiation, proliferation, and metastasis. We aimed to evaluate the association of the preoperative systemic immune-inflammation index (SII) with the clinical outcomes of patients diagnosed with bladder cancer and who underwent radical cystectomy (RC).

**Materials and methods:**

Data from patients diagnosed with bladder cancer and who underwent RC from December 2010 to May 2020 in West China Hospital were retrospectively collected according to the inclusion and exclusion criteria. Patients were divided into a low-SII group and a high-SII group according to the SII level. Survival outcomes were obtained during follow-up. The primary endpoints were overall survival (OS) and recurrence-free survival (RFS). Cox proportional hazard models were performed to estimate the effect of SII on OS and RFS and control for potential confoundings. Subgroup analyses were conducted, and the log likelihood ratio test was used to inspect the interaction.

**Results:**

A total of 725 patients who underwent RC were ultimately involved in this study. Of these patients, 621 (85.66%) were men and 104 (14.34%) were women. The median age was 65 years. The median follow-up was 36 months for OS and 33.6 months for RFS. The optimal cutoff value was identified as 554.23 × 10^9^/l. A total of 467 (64.41%) patients were divided into the low-SII group (SII <554 × 10^9^/l), and 258 (35.59%) patients were divided into the high-SII group (SII ≥554 × 10^9^/l) accordingly. Multivariable Cox proportional hazard regression demonstrated that a high SII was an independent prognostic factor for worse OS (HR: 1.69 95% CI: 1.02–2.81, P = 0.0436) and RFS (HR: 1.88, 95% CI: 1.09–3.24, P = 0.0229) in NMIBC patients. A high SII was found to be an independent prognostic factor for worse RFS in patients with HBP (HR: 2.11, 95% CI: 1.34–3.30, P = 0.0012), with DM (HR: 3.76, 95% CI: 1.73–8.15, P = 0.0008), and without PNI (HR: 1.32, 95% CI: 1.04–1.69, P = 0.0238).

**Conclusions:**

The SII was a potential prognostic predictor for bladder cancer patients who underwent RC. Further prospective multicenter investigations are warranted.

## Introduction

Bladder cancer is the sixth most commonly diagnosed cancer in men and dropped to 10th when considering both genders, with estimated 573,278 new cases and 212,536 new deaths worldwide in 2020 (1). Men are more affected than women (approximately 3:1). The median age of diagnosis was 69 years in men and 71 years in women (2). According to the depth of invasion, bladder cancer can be categorized into muscle­invasive bladder cancer (MIBC) and non-muscle­invasive bladder cancer (NMIBC). Tumors isolated to the urothelium (stage Ta) and the lamina propria (stage T1) are considered NMIBC, which accounts for 75% of the newly diagnosed cases. For NMIBC, all of these tumors can be treated by primary transurethral resection of the bladder (TURB) followed by intravesical instillations at the physician’s discretion. NMIBC can be stratified by clinical and pathological risk factors, for those who are classified into the high-/very high-risk group of disease progression or failure to Bacillus Calmette–Guerin (BCG) instillations are recommended to have radical cystectomy (RC). Tumors that invade the detrusor muscle (stage≥T2) are considered MIBC and are more likely to metastasize to lymph nodes or other organs; approximately 25% of patients are diagnosed with MIBC or already have metastasis at the first diagnosis (3, 4). For MIBC, the first treatment option is RC. However, nearly 50% of patients who undergo RC have distant recurrence (5). To date, limited preoperative biomarkers that are easy to obtain and cost-effective have been used to predict the prognosis in the clinic (6). Searching more accurate and convenient biomarkers could facilitate patient counseling, as well as treatment and follow-up strategies.

Inflammation is considered as a hallmark feature of the initiation and promotion of carcinogenesis (7). Inflammation found at the local site of the tumor is commonly considered to be local immune response, which consists of immune cells, inflammatory protein mediators, and cytokines and constructs the local tumor microenvironment. Tumor-derived cytokines and mediators are secreted into the systemic circulation to mediate communication with distant sites. Systemic inflammation consists of circulating cytokines, circulating immune cells, and inflammatory-associated proteins, which are crucial for tumor metastasis and have crosstalk with the local tumor immune response, are detectable, and can frequently mark the presence and progression of cancer (8, 9). Therefore, biomarkers derived from blood samples that are routinely recorded are arousing considerable interest. Here, we introduce the systemic immune-inflammation index (SII), which is a combination of neutrophil, lymphocyte, and platelet counts in blood. Also, it has been widely investigated and shown to be a useful prognostic indicator in various solid tumors including hepatocellular carcinoma (10), small cell lung cancer (11), gastroesophageal adenocarcinoma (12, 13), renal cell carcinoma (14), and prostate cancer (15, 16).

The purpose of this study was to examine the prognostic value of the SII in patients with bladder cancer who underwent RC. In addition, we determined which population could benefit from the assessment of the SII for predicting survival outcome. This can help facilitate clinicians in administering further adjuvant therapies and a having a closer follow-up.

## Methods

### Patients

Patients with NMIBC who are at a very high risk of disease progression and failure to BCG instillations or patients with MIBC are recommended to receive RC. Open, laparoscopic, or robot-assisted RC was performed through standard procedures, with or without pelvic lymph node dissection (PLND). We retrospectively collected data from patients who were diagnosed with bladder cancer and underwent RC from December 2010 to May 2020 in West China Hospital. The exclusion criteria are as follows: 1) preoperative blood examination data were unavailable; 2) did not receive postoperative follow-up at our institutions; 3) missing pathological T stage; 4) with metastasis before operation; 5) main histology were not urothelial carcinoma; 6) with other tumors. This retrospective cohort study followed the Declaration of Helsinki and was approved by the Ethics Committee of West China Hospital, Sichuan University.

### Systemic immune-inflammation index (SII)

Preoperative blood examination data were collected from each patient’s electronic medical record. The SII was calculated as follows: SII = neutrophil count (10^9^/L) × platelet count (10^9^/L)/lymphocyte count (10^9^/L). Time-dependent receiver operating characteristic (ROC) curves for overall survival and recurrence-free survival prediction were performed, and the maximum Youden index which was calculated as “sensitivity + specificity – 1” was used to select the optimal cutoff value. According to the SII levels, patients were divided into two groups: the low-SII group and the high-SII group.

### Covariates

The clinicopathological data were retrospectively collected, including gender, age, BMI, smoking history, high blood pressure (HBP), diabetes mellitus (DM), tumor diameter, tumor number, tumor grade, pT stage, pN stage, concomitant CIS, concomitant variant histology, presence of positive surgical margins (PSM), peripheral nerve invasion (PNI), and lymphovascular invasion (LVI). All specimens were staged according to the American Joint Committee on Cancer Staging Manual (8th edition) TNM classification and graded according to the 1973 World Health Organization (WHO) grading system. Neoadjuvant therapy or adjuvant therapy was administered according to the surgeon’s discretion combined with the patients’ consideration. Operation information, including methods, pelvic lymph node dissection (PLND), and urinary diversion, was also documented.

### Follow-up and outcome

All patients were recommended to have CT scans of the abdomen and pelvis every 3 months for the first year postoperatively, every 6 months for the next 2 years, and once a year thereafter. Follow-up methods mainly included phone encounters and outpatient interviews. Overall survival (OS) was defined as the time interval from the time of operation to the time of death or final contact. Recurrence-free survival (RFS) was defined as the time interval from the time of operation to the time of recurrence, including local/urothelial recurrence and distant metastasis.

### Statistical analysis

Categorical variables are presented as numbers and proportions, and continuous variables are presented as the mean with standard deviation (SD). The Kruskal–Wallis test and Fisher’s exact test were used to determine if there were any statistically significant differences. Univariate or multivariate logistic regression was performed to explore the associations between preoperative SII and adverse pathological outcomes. The Kaplan–Meier method was used to estimate OS and RFS, and the log-rank test was used to assess differences between survival. Univariable Cox proportional hazard models were performed to estimate the effect of each predictor on OS and RFS. Multivariable Cox proportional hazard models were carried out to control potential confoundings and determine the independent predictors of OS and RFS after RC. We adjusted variables for the feature that, when they are added to the model, changed the matched hazard ratio by at least 10%. Subgroup analyses were conducted, and the log-likelihood ratio test was used to inspect the interaction. All analyses were performed using the statistical software packages R (http://www.R-project.org, The R Foundation) and EmpowerStats (http://www.empowerstats.com, X&Y Solutions, Inc., Boston, MA, version 4.2.1). All tests were two sided, and P values<0.05 were considered statistically significant.

## Results

### Patient characteristics

**Figure 1:**
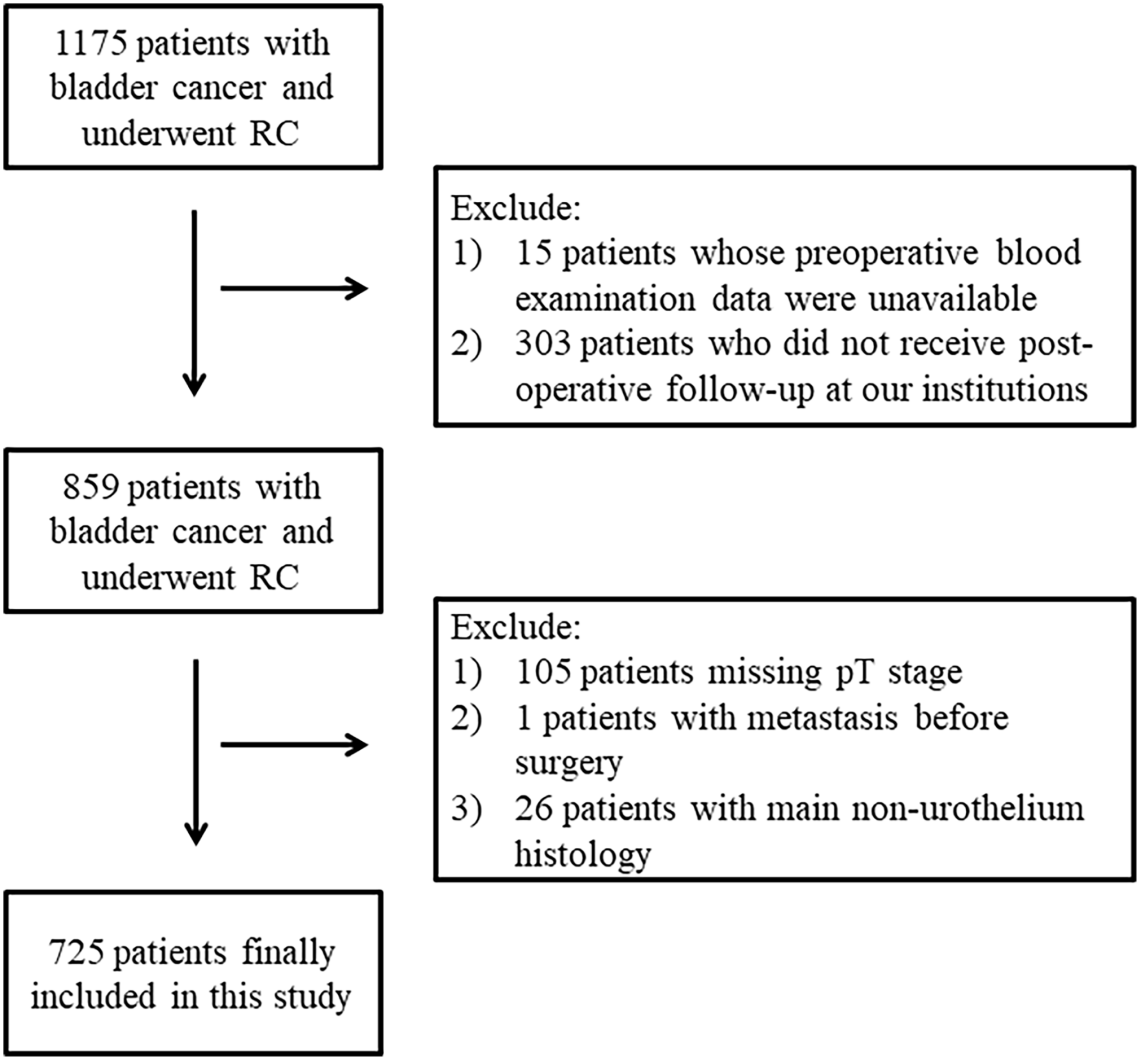
Flow diagram of included participants.

A total of 725 patients who underwent RC were ultimately involved in this study (**Figure 1**). Of these patients, 621 (85.66%) were men and 104 (14.34%) were women. The median age was 65 years (interquartile range: 59–72 years), and the average BMI was 23.25 ± 2.72 kg/m^2^. There were 208 (28.73%) patients who had high blood pressure, 103 (14.23%) of them had historically diagnosed diabetes mellitus, and 55.03% had smoking history. A total of 6.09% of them received neoadjuvant therapy, and 14.7% of patients received adjuvant therapy including radiotherapy (3.47%), chemotherapy (9.99%), and both (1.25%) postoperatively. Most patients underwent open RC (56.91%), 39.50% underwent laparoscopic RC, and 3.45% underwent robot-assisted RC. The majority of urinary diversion methods used were ileal conduit (86.58%), cutaneous ureterostomy (10.79%), and orthotopic neobladder (2.63%) in a small number of patients. A total of 64.97% of the patients experienced pelvic lymph node dissection. With regard to pathological characteristics, 480 (66.21%) patients were diagnosed with ≥T2 stage and 123 (16.7%) of these patients had lymph node invasion. PSM, PNI, and LVI was present in 8.00%, 11.62%, and 21.79% of these patients, respectively. In addition, 623 patients were diagnosed as high grade (88.87%). There were 188 patients (26.55%) with variant histology, and 20 patients had concomitant CIS. A total of 87.17% of these patients had a tumor diameter ≥3cm, and 42.34% of them had multiple tumors (**Table 1**).

**Table 1:** Baseline characteristics of included participants.

Characteristics	Overall (n = 725)	SII		P value
		Low (n = 467)	High (n = 258)	
**Age (median (IQR), years)**	65.00 (59.00-72.00)	65.00 (58.00-71.50)	66.00 (60.00-72.00)	0.213
**BMI (mean ± SD, kg/m^2^)**	23.25 ± 2.72	23.30 ± 2.72	23.16 ± 2.72	0.490
**SII (median (IQR) × 10** ^9^ **/L)**	424.13 (282.55-714.84)	323.55 (237.36-411.25)	830.76 (681.95-1236.50)	<0.001
**n (%)**				
**Age (years)**				0.106
<65	344 (47.45%)	232 (49.68%)	112 (43.41%)	
≥65	381 (52.55%)	235 (50.32%)	146 (56.59%)	
**Gender**				0.375
Male	621 (85.66%)	396 (84.80%)	225 (87.21%)	
Female	104 (14.34%)	71 (15.20%)	33 (12.79%)	
**Smoking history**				0.550
No	324 (44.69%)	205 (43.99%)	119 (46.30%)	
Yes	399 (55.03%)	261 (56.01%)	138 (53.70%)	
**Pathological T stage**				<0.001
T0	27 (3.72%)	18 (3.85%)	9 (3.49%)	
T1	216 (29.79%)	160 (34.26%)	56 (21.71%)	
T2	163 (22.48%)	117 (25.05%)	46 (17.83%)	
T3	212 (29.24%)	115 (24.63%)	97 (37.60%)	
T4	105 (14.48%)	55 (11.78%)	50 (19.38%)	
Tis	2 (0.28%)	2 (0.43%)	0 (0.00%)	
**Pathological N stage**				0.023
N0	348 (48.00%)	234 (50.11%)	114 (44.19%)	
N1	42 (5.79%)	22 (4.71%)	20 (7.75%)	
N2	79 (10.90%)	40 (8.57%)	39 (15.12%)	
N3	2 (0.28%)	1 (0.21%)	1 (0.39%)	
Nx	254 (35.03%)	170 (36.40%)	84 (32.56%)	
**Grade**				0.352
Low	78 (11.13%)	54 (11.95%)	24 (9.64%)	
High	623 (88.87%)	398 (88.05%)	225 (90.36%)	
**Variant histology**				0.028
No	520 (73.45%)	348 (76.15%)	172 (68.53%)	
Yes	188 (26.55%)	109 (23.85%)	79 (31.47%)	
**Concomitant CIS**				
No	705 (97.24%)	455 (97.43%)	250 (96.90%)	0.676
Yes	20 (2.76%)	12 (2.57%)	8 (3.10%)	
**Positive surgical margins**				0.450
No	667 (92.00%)	427 (91.43%)	240 (93.02%)	
Yes	58 (8.00%)	40 (8.57%)	18 (6.98%)	
**Peripheral nerve invasion**				0.223
No	639 (88.38%)	416 (89.46%)	223 (86.43%)	
Yes	84 (11.62%)	49 (10.54%)	35 (13.57%)	
**Lymphovascular invasion**				0.203
No	567 (78.21%)	372 (79.66%)	195 (75.58%)	
Yes	158 (21.79%)	95 (20.34%)	63 (24.42%)	
**Tumor diameter**				0.005
<3cm	93 (12.83%)	72 (15.42%)	21 (8.14%)	
≥3cm	632 (87.17%)	395 (84.58%)	237 (91.86%)	
**Tumor number**				0.054
Unifocal	418 (57.66%)	257 (55.03%)	161 (62.40%)	
Multifocal	307 (42.34%)	210 (44.97%)	97 (37.60%)	
**Neoadjuvant therapy**				0.830
No	678 (93.91%)	436 (93.76%)	242 (94.16%)	
Yes	44 (6.09%)	29 (6.24%)	15 (5.84%)	
**Adjuvant therapy**				0.220
None	615 (85.30%)	392 (84.48%)	223 (86.77%)	
Radiotherapy	25 (3.47%)	13 (2.80%)	12 (4.67%)	
Chemotherapy	72 (9.99%)	52 (11.21%)	20 (7.78%)	
Radio+Chemo	9 (1.25%)	7 (1.51%)	2 (0.78%)	
**Operation method**				0.062
Laparoscopic RC	286 (39.50%)	197 (42.27%)	89 (34.50%)	
Open RC	412 (56.91%)	249 (53.43%)	163 (63.18%)	
Robot-assisted RC	26 (3.59%)	19 (4.28%)	6 (2.33%)	
**Urinary diversion**				0.828
Ileal Conduit	626 (86.58%)	405 (86.91%)	221 (85.99%)	
Cutaneous Ureterostomy	78 (10.79%)	50 (10.73%)	28 (10.89%)	
Orthotopic Neobladder	19 (2.63%)	11 (2.36%)	8 (3.11%)	
**Pelvic lymph node dissection**				0.299
No	254 (35.03%)	170 (36.40%)	84 (32.56%)	
Yes	471 (64.97%)	297 (63.60%)	174 (67.44%)	
**High blood pressure**				0.886
No	516 (71.27%)	332 (71.09%)	184 (71.60%)	
Yes	208 (28.73%)	135 (28.91%)	73 (28.40%)	
**Diabetes mellitus**				0.749
No	621 (85.77%)	402 (86.08%)	219 (85.21%)	
Yes	103 (14.23%)	65 (13.92%)	38 (14.79%)	

IQR, interquartile range; SD, standard deviation; BMI, body mass index; SII, systemic immune-inflammation index; CIS, carcinoma *in situ*.

### The optimal cutoff values of the SII

The median SII of this population was 424.13 × 10^9^/l (interquartile range: 282.55–714.84 × 10^9^/l). The areas under the curves were 0.523 and 0.531 for predicting OS and RFS, respectively. The corresponding optimal cutoff value was identified as 554.23 × 10^9^/l for both OS and RFS to yield the maximum Youden index (**Supplemental Figure 1**). Accordingly, 467 (64.41%) patients were divided into the low-SII group (SII <554 × 10^9^/l) and 258 (35.59%) patients were divided into the high-SII group (SII ≥554 × 10^9^/l). As shown in **Table 1**, there were no statistically significant differences between the two groups regarding age, BMI, gender, grade, concomitant CIS, tumor number, PSM, PNI, LVI, neoadjuvant therapy, adjuvant therapy and operation method, urinary diversion method, PLND, LNY, smoking history, HBP, and DM. However, significant differences were found between the two groups in pT, pN stage, concomitant variant histology, and tumor diameter (P < 0.05).

### The associations between preoperative SII and pathological features

Logistic regression analyses were performed to explore the associations between preoperative SII and adverse pathological outcomes (**Supplemental Table 1**). In both univariate and multivariate analyses, a high SII was positively associated with larger tumor size (diameter ≥3 cm, OR: 2.05, 95% CI: 1.22–3.43, P = 0.0065), high pT stage (OR: 1.88, 95% CI: 1.33–2.65, P = 0.0003), and variant histology (OR: 1.44, 95% CI: 1.02–2.04, P = 0.0380).

### The relationship between preoperative SII and OS

**Figure 2:**
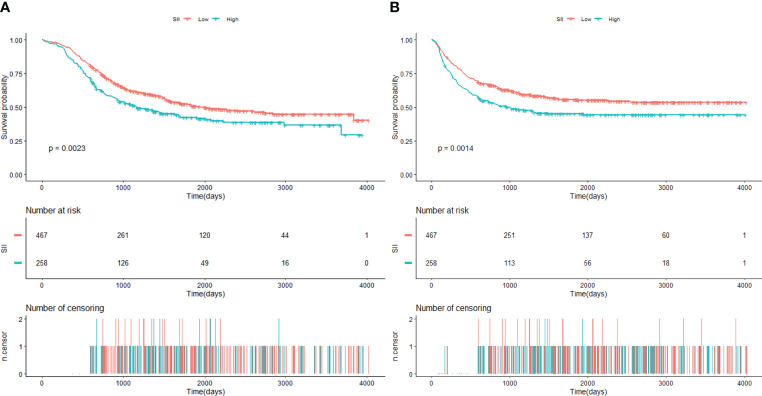
**(A)** Kaplan–Meier estimate of overall survival (OS) of patients according to SII level. **(B)** Kaplan–Meier estimate of recurrence-free survival (RFS) of patients according to SII level (SII cutoff value: 554.23 × 10^9^/L). SII, systemic immune-inflammation index.

The median follow-up time was 1,079 days (36 months, interquartile range: 617–1,092 days). A total of 364 (50.21%) patients died during this time. The 5-year OS rate in the low-SII group and the high-SII group were 52.32% (95% CI: 47.79%–57.29%) and 40.81% (95% CI: 35.04%–47.53%), respectively. As Kaplan–Meier curves showed, the high-SII group was associated with worse OS compared with patients in the low-SII group (log-rank P = 0.0023, **Figure 2A**).

Univariable Cox proportional hazard regression revealed that a high preoperative SII predicted poor overall survival (HR: 1.38, 95% CI: 1.12–1.71, P = 0.0025, **Table 2**). According to confounding selection criteria, age, pT stage, pN stage, gender, adjuvant therapy, PSM, and LVI were included in the multivariate model. However, after adjusting for all these covariates, the HR was attenuated in multivariate analyses (HR: 1.15, 95% CI: 0.92–1.43, P = 0.2242, **Table 3**).

**Table 2:** Univariate analyses of the association between baseline characteristics and overall survival (OS) and recurrence-free survival (RFS).

	OS		RFS	
	HR (95% CI)	P value	HR (95% CI)	P value
**Age (years)**	1.02 (1.01, 1.03)	<0.0001	1.01 (1.00, 1.02)	0.0364
**Age (years)**				
<65	1.0		1.0	
≥65	1.45 (1.17, 1.78)	0.0005	1.34 (1.08, 1.66)	0.0079
**BMI (kg/m^2^)**	0.99 (0.95, 1.03)	0.6851	0.99 (0.95, 1.03)	0.5125
**Pathological T stage**				
T0	1.0		1.0	
T1	1.84 (0.74, 4.56)	0.1890	4.24 (1.04, 17.36)	0.0442
T2	3.04 (1.23, 7.51)	0.0162	7.34 (1.80, 29.94)	0.0054
T3	6.08 (2.49, 14.84)	<0.0001	13.17 (3.26, 53.24)	0.0003
T4	6.83 (2.76, 16.93)	<0.0001	17.22 (4.23, 70.19)	<0.0001
**Pathological N stage**				
N0	1.0		1.0	
N1	4.53 (3.11, 6.62)	<0.0001	3.72 (2.51, 5.54)	<0.0001
N2	4.37 (3.24, 5.89)	<0.0001	4.01 (2.96, 5.43)	<0.0001
N3	9.20 (2.26, 37.45) 0.0019		6.85 (1.68, 27.88) 0.0072	
Nx	1.62 (1.27, 2.06)	0.0001	1.50 (1.17, 1.93)	0.0015
**Grade**				
Low	1.0		1.0	
High	2.94 (1.87, 4.61)	<0.0001	3.43 (2.04, 5.76)	<0.0001
**Variant histology**				
No	1.0		1.0	
Yes	1.55 (1.24, 1.94)	0.0001	1.48 (1.17, 1.86)	0.0010
**Concomitant CIS**				
No	1.0		1.0	
Yes	0.83 (0.41, 1.68)	0.6097	0.69 (0.33, 1.46)	0.3354
**Tumor diameter**				
<3 cm	1.0		1.0	
≥3 cm	1.37 (0.98, 1.92)	0.0661	1.34 (0.95, 1.89)	0.0980
**Tumor number**				
Unifocal	1.0		1.0	
Multifocal	0.87 (0.71, 1.07)	0.1985	0.84 (0.68, 1.04)	0.1152
**Positive surgical margins**				
No	1.0		1.0	
Yes	1.91 (1.39, 2.64)	<0.0001	1.77 (1.27, 2.48)	0.0008
**Peripheral nerve invasion**				
No	1.0		1.0	
Yes	1.91 (1.44, 2.54)	<0.0001	1.93 (1.45, 2.58)	<0.0001
**Lymphovascular invasion**				
No	1.0		1.0	
Yes	2.08 (1.66, 2.61)	<0.0001	2.01 (1.60, 2.54)	<0.0001
**Neoadjuvant therapy**				
No	1.0		1.0	
Yes	0.71 (0.42, 1.19)	0.1929	0.81 (0.49, 1.31)	0.3862
**Adjuvant therapy**				
None	1.0		1.0	
Radiotherapy	1.29 (0.77, 2.17)	0.3317	1.63 (0.97, 2.75)	0.0650
Chemotherapy	1.49 (1.10, 2.02)	0.0108	2.13 (1.59, 2.86)	<0.0001
Radio+Chemo	1.78 (0.79, 3.99)	0.1635	2.22 (0.99, 4.99)	0.0535
**Operation method**				
Laparoscopic RC	1.0		1.0	
Open RC	1.20 (0.96, 1.50)	0.1026	1.29 (1.03, 1.62)	0.0274
Robot-assisted RC	1.01 (0.53, 1.93) 0.9690	0.9690	1.04 (0.54, 1.98)	0.9062
**Urinary diversion**				
Ileal conduit	1.0		1.0	
Cutaneous Ureterostomy	1.80 (1.32, 2.45)	0.0002	1.31 (0.94, 1.82)	0.1138
Orthotopic neobladder	0.74 (0.38, 1.43)	0.3649	0.65 (0.31, 1.37)	0.2530
**Pelvic lymph node dissection**				
No	1.0		1.0	
Yes	0.95 (0.77, 1.18)	0.6550	1.00 (0.80, 1.25)	0.9845
**Smoking history**				
No	1.0		1.0	
Yes	0.88 (0.72, 1.08)	0.2300	0.85 (0.69, 1.06)	0.1432
**High blood pressure**				
No	1.0		1.0	
Yes	0.90 (0.71, 1.13)	0.3687	0.93 (0.73, 1.18)	0.5370
**Diabetes mellitus**				
No	1.0		1.0	
Yes	0.65 (0.47, 0.90)	0.0094	0.76 (0.55, 1.06)	0.1039
**SII**				
Low	1.0		1.0	
High	1.38 (1.12, 1.71)	0.0025	1.42 (1.14, 1.76)	0.0015

BMI, body mass index; SII, systemic immune-inflammation index; CIS, carcinoma in situ; HR, hazards ratio; CI, confidence interval.

**Table 3:** Multivariate Cox regression analyses estimating the influence of SII on overall survival (OS) and recurrence-free survival (RFS).

	Adjusted HR (95% CI)	P value	P for interaction
**OS** ^a^			
**Total**	1.14 (0.91, 1.42)	0.2484	
Pathological T stage			
T0TisTaT1	1.69 (1.02, 2.81)	0.0436	0.1695
≥T2	1.10 (0.87, 1.40)	0.43	
**RFS** ^b^			
**Total**	1.18 (0.94, 1.48)	0.1488	
Pathological T stage			
T0TisTaT1	1.88 (1.09, 3.24)	0.0229	0.1755
≥T2	1.17 (0.91, 1.49)	0.2168	
HBP			
No	0.93 (0.70, 1.22)	0.5876	0.0024
Yes	2.11 (1.34, 3.30)	0.0012	
DM			
No	1.04 (0.82, 1.34)	0.7296	0.014
Yes	3.76 (1.73, 8.15)	0.0008	
PSM			
No	1.11 (0.87, 1.41)	0.4022	0.0411
Yes	2.05 (0.83, 5.03)	0.1188	
PNI			
No	1.32 (1.04, 1.69)	0.0238	0.0241
Yes	0.79 (0.42, 1.49)	0.4724	

SII, systemic immune-inflammation index; HBP, high blood pressure; DM, diabetes mellitus; PSM, positive surgical margins; PNI, peripheral nerve invasion; HR, hazards ratio; CI, confidence interval.

a Adjusted for age, pT stage, pN stage, gender, adjuvant therapy, PSM, LVI.

b Adjusted for age, gender, adjuvant therapy, pT, pN, PSM.

We further examined the association between SII and OS stratified by different clinicopathological characteristics and various treatment regimens and surgical procedures (**Supplemental Figure 2A**). Subgroup analyses indicated that high SII was an independent prognostic factor for worse OS in patients with pT0TisT1 stage disease (HR: 1.69 95% CI: 1.02–2.81, P = 0.0436, **Table 3**). In all subgroups, the tests for interactions were not statistically significant.

### The relationship between preoperative SII and RFS

The median follow-up time for RFS was 1,007 days (33.6 months, interquartile range: 329–2,065 days). A total of 340 (46.9%) patients experienced recurrence in the follow-up period. The 5-year RFS rate was 55.94% (95% CI: 51.51%–60.74%) in the low-SII group and 43.80% (95% CI: 38.05%–50.41%) in the high-SII group. Kaplan–Meier curves showed that the high group was associated with decreased RFS (log-rank P = 0.0014, **Figure 2B**).

Univariable Cox proportional hazard regression demonstrated that a high SII predicted poor RFS (HR: 1.42 95% CI: 1.14–1.76, P = 0.0015, **Table 2**). In multivariate analyses, after we adjusted for age, gender, adjuvant therapy, pT, pN, PSM, and PNI, the HR was attenuated (HR: 1.19, 95% CI: 0.95–1.49, P = 0.1403, **Table 3**).

In subgroup analyses (**Supplemental Figure 2B**), a high SII was found to be an independent prognostic factor for worse RFS in patients with HBP (HR: 2.11, 95% CI: 1.34–3.30, P = 0.0012), with DM (HR: 3.76, 95% CI: 1.73–8.15, P = 0.0008), without PNI (HR: 1.32, 95% CI: 1.04–1.69, P = 0.0238), and with pT0TisT1 stage tumor (HR: 1.88, 95% CI: 1.09–3.24, P=0.0229, **Table 3**). Tests for interactions were statistically significant for HBP, DM, PSM, and PNI (all P < 0.05), indicating that these factors may act as modifiers in the relationship between preoperative SII and RFS. The effects of SII on RFS were opposite in different aspects of modifiers. For patients with high blood pressure, diabetes, and PSM, an elevated SII was strongly associated with worse RFS, whereas in patients without these features, the HRs were largely attenuated and not statistically significant. In contrast, for patients with PNI, a high SII was associated with favorable RFS (HR: 0.79, 95% CI: 0.42–1.49, P = 0.4724), whereas for patients without PNI, the effect size was reported above and consistent with the overall results.

## Discussion

In this study, we evaluated the prognostic potential of the SII for patients with bladder cancer who underwent RC in a large cohort and revealed that patients with an increased preoperative SII had inferior OS and RFS outcomes. In the univariate Cox proportional hazard model, a higher SII was significantly associated with worse OS and RFS. Unfortunately, in multivariate analyses, after adjusting for potential cofounders, the SII did not remain a statistically significant independent predictor of OS and RFS for the whole population. Specifically, subgroup analyses demonstrated that the SII could independently predict OS and RFS for NMIBC patients. Interestingly, the SII was found to be independently correlated with RFS for patients with HBP and DM and without PNI, HBP, and DM. Furthermore, PSM and PNI might be modifiers for the association of SII and RFS. Univariate and multivariate logistic regressions indicated that a high SII is positively associated with poor pathological outcomes, including larger tumor size, high pT stage, and variant histology.

Recently, several studies have investigated the prognostic value of SII in bladder cancer and remain controversial. Yılmaz (17) performed multivariate analyses and found that the SII was not an independent prognostic factor in MIBC. Nevertheless, Gorgel et al. (18–20) claimed that the SII could be an independent prognostic factor in MIBC patients who underwent RC. Researchers investigated the associations between SII and high-risk NMIBC patients who received intravesical instillation of BCG after TURBT and revealed that the preoperative SII was an independent predictor for OS, CSS (21), and RFS (22). For NMIBC, distinct from other studies, we collected the SII after TURBT and intravesical instillations, which still remained an indicator for survival after subsequent RC, implying that the SII could predict prognosis regardless of the time point during treatment periods. Li and colleagues (23) conducted a meta-analysis involving 7,087 patients diagnosed with bladder cancer who underwent RC or TURBT. The pooled results indicated that an elevated SII was significantly related to worse OS, CSS, and RFS.

The inflammatory tumor microenvironment and related host systemic inflammation might play crucial roles in tumor carcinogenesis, proliferation, invasion, and dissemination and alter responses to hormones and chemotherapeutic agents (24, 25). Numerous researchers have examined the prognostic value of markers including C-reactive protein (CRP) (26), neutrophil-to-lymphocyte ratio (NLR), and platelet-to-lymphocyte ratio (PLR), which reflect host inflammatory status and local immune response status. Neutrophils were reported to be involved in tumor progression by promoting genetic instability and stimulating angiogenesis (27), while platelets activated by tumor cells would in turn facilitate tumor cell dissemination by inducing an opening of the endothelial barrier to allow transendothelial migration (28). Lymphocytes are generally known to play a critical role in antitumor immunity. The SII is a novel marker that integrates neutrophils, platelets, and lymphocytes from blood samples. Therefore, the SII is potentially an easily accessible and inexpensive prognostic biomarker that might be used in different clinical scenarios including improving risk stratification, monitoring disease progression, and predicting treatment response.

Our study has some limitations: first of all, owing to the single-center retrospective design, selection bias was inevitable and external validity was limited. Secondly, some postoperative treatment information and survival outcomes during follow-up were obtained from telephone conversations with patients’ relatives, which might cause recall bias. Thirdly, for NMIBC patients who underwent RC, preoperative use of intravesicle therapy, which might alter the level of SII and influence the oncological outcome, was not controlled. Finally, the use of drugs for patients with HBP, DM, or other comorbidities that might influence prognosis was not recorded and taken into account.

## Conclusion

Taken together, the novel, recently developed immunological marker SII was a potential prognostic predictor for bladder cancer patients who underwent RC. An elevated preoperative SII was associated with adverse pathological features and worse survival outcomes. Furthermore, the SII was an independent predictor of OS and RFS for NMIBC patients and could independently predict RFS for patients with either HBP or DM and patients without PNI. Further investigations on the application of the SII in clinical practice are warranted. Prospective multicenter analyses are needed in the future.

## Data availability statement

The raw data supporting the conclusions of this article will be made available by the authors, without undue reservation.

## Ethics statement

The studies involving human participants were reviewed and approved by The Ethics Committee of West China Hospital, Sichuan University. The patients/participants provided their written informed consent to participate in this study.

## Author contributions

Manuscript writing and statistical analysis: SZ, JJ. Data collection: XZ, JP, HX. Conception and literature research: YL, DJ, WL. Manuscript review and editing: PT, TL, LY. Financial support: TL, QW. All authors contributed to the article and approved the submitted version.

## Funding

This work was supported by the National Natural Science Foundation of China (Grant No. 82170784).

## Conflict of interest

The authors declare that the research was conducted in the absence of any commercial or financial relationships that could be construed as a potential conflict of interest.

## Publisher’s note

All claims expressed in this article are solely those of the authors and do not necessarily represent those of their affiliated organizations, or those of the publisher, the editors and the reviewers. Any product that may be evaluated in this article, or claim that may be made by its manufacturer, is not guaranteed or endorsed by the publisher.

## Supplementary material

Supplementary Figure 1 Time-dependent ROC curve for overall survival (OS) **(A)** and recurrence-free survival (RFS) **(B)**. ROC, receiver-operating characteristic; AUC: area under the curve.

Supplementary Figure 2
**(A)** Subgroup analyses for overall survival (OS) based on SII levels. Each stratification adjusted for all the factors including age, gender, pT stage, pN stage, adjuvant therapy, PSM, LVI, except for stratification factor itself. **(B)** Subgroup analyses for recurrence-free survival (RFS) based on SII levels. Each stratification adjusted for all the factors including age, gender, adjuvant therapy, pT, pN, PSM, PNI, except for stratification factor itself. SII, systemic immune-inflammation index; BMI, body mass index; HR: hazards ratio; HBP: high blood pressure. DM: diabetes mellitus; PSM: positive surgical margins PNI: peripheral nerve invasion; LVI: lymphovascular invasion; CI, confidence interval.
