# Supplementary figures and images for: The prognostic value of the systemic immune-inflammation index for patients with bladder cancer after radical cystectomy
Source: Front Immunol. 2022 Nov 29;13:1072433. doi: 10.3389/fimmu.2022.1072433 (PMC9744948; doi:10.3389/fimmu.2022.1072433)

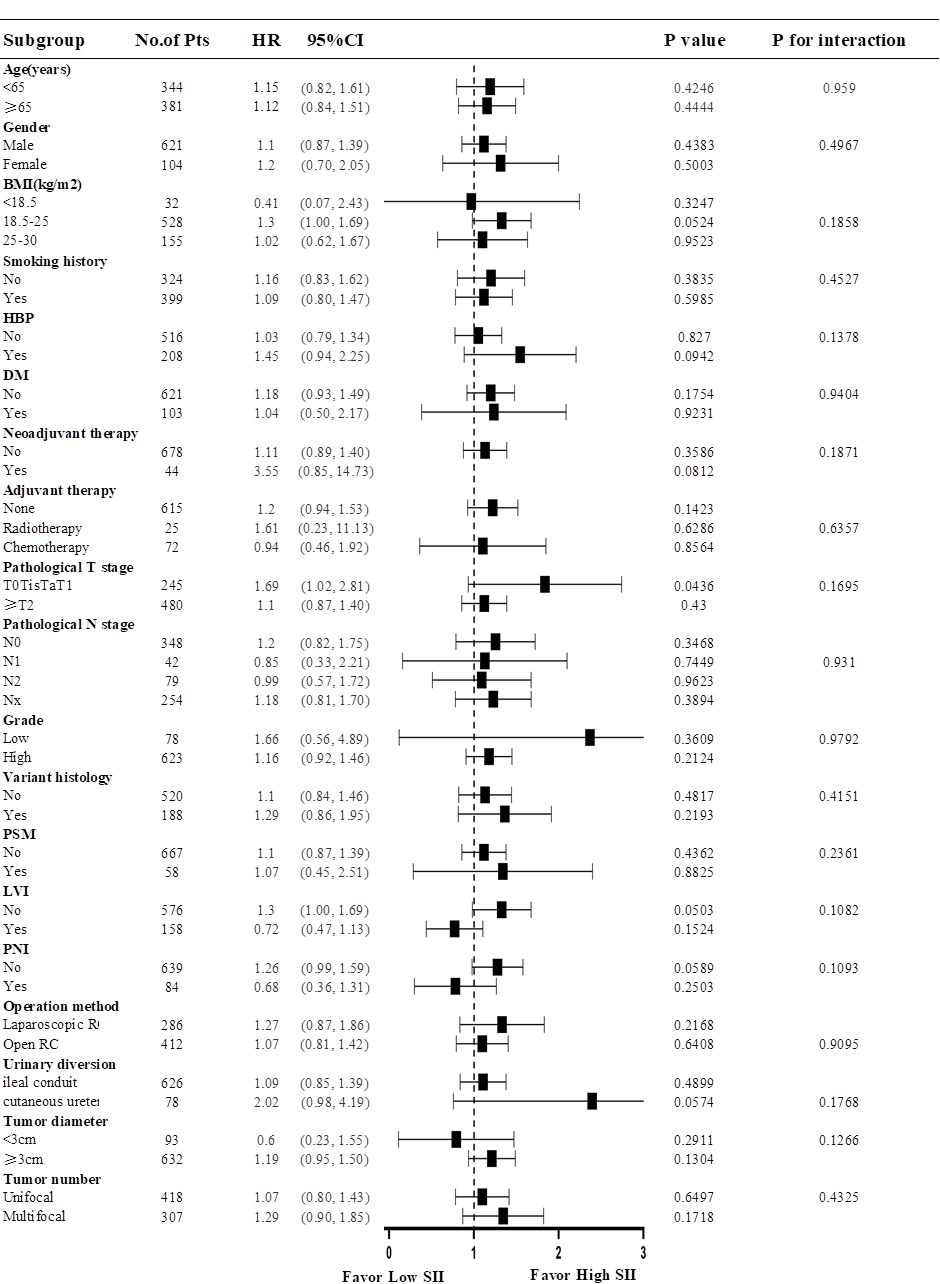

Supplement: Supplementary file 3 [file Image_3.tif]

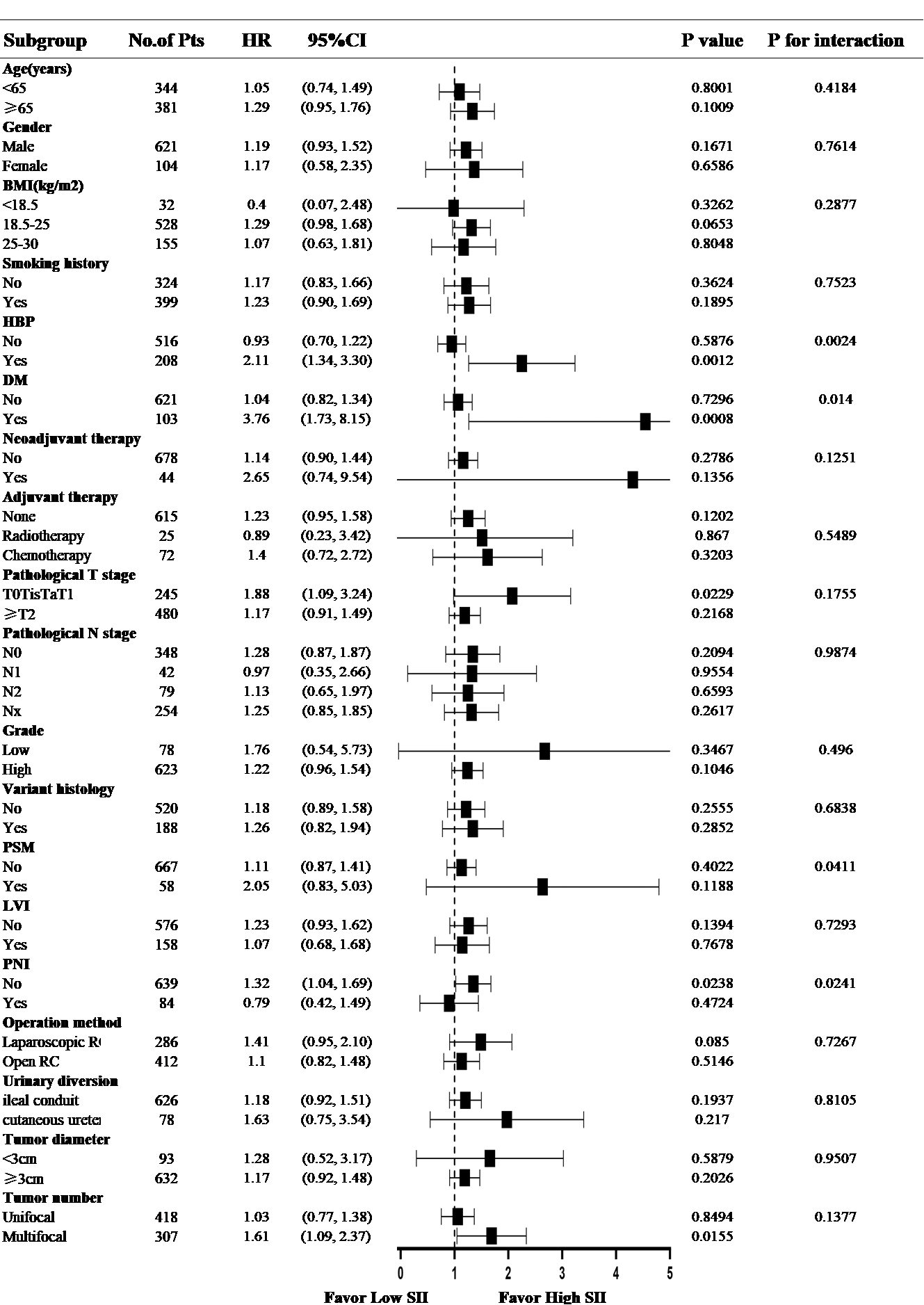

Supplement: Supplementary file 4 [file Image_4.tif]
