# Supplementary material for: The prognostic value of the systemic immune-inflammation index for patients with bladder cancer after radical cystectomy
Source: Front Immunol. 2022 Nov 29;13:1072433. doi: 10.3389/fimmu.2022.1072433 (PMC9744948; doi:10.3389/fimmu.2022.1072433)
Supplement: Supplementary file 5 [file Table_1.docx]

**Supplemental Table 1.** The associations between preoperative SII and pathologic features.

| Pathologic Features | Univariate | | Multivariate | |
| --- | --- | --- | --- | --- |
|  | OR (95%CI) | P value | OR (95%CI) | P value |
| Tumor Diameter |  |  |  |  |
| <3cm | 1.0 |  | 1.0 |  |
| ≥3cm | 2.06 (1.23, 3.43) | **0.0058** | 2.05 (1.22, 3.43) | **0.0065** |
| Tumor Number |  |  |  |  |
| Unifocal | 1.0 |  | 1.0 |  |
| Multifocal | 0.74 (0.54, 1.01) | 0.0548 | 0.74 (0.54, 1.01) | 0.0587 |
| Pathological T Stage |  |  |  |  |
| T0TisTaT1 | 1.0 |  | 1.0 |  |
| ≥T2 | 1.86 (1.33, 2.61) | **0.0003** | 1.88 (1.33, 2.65) | **0.0003** |
| Grade |  |  |  |  |
| Low | 1.0 |  | 1.0 |  |
| High | 1.27 (0.77, 2.11) | 0.3532 | 1.24 (0.74, 2.08) | 0.4172 |
| Variant Histology |  |  |  |  |
| No | 1.0 |  | 1.0 |  |
| Yes | 1.47 (1.04, 2.07) | **0.0284** | 1.44 (1.02, 2.04) | **0.0380** |
| Concomitant CIS |  |  |  |  |
| No | 1.0 |  | 1.0 |  |
| Yes | 1.21 (0.49, 3.01) | 0.6763 | 1.22 (0.49, 3.04) | 0.6651 |
| Positive Surgical Margins |  |  |  |  |
| No | 1.0 |  | 1.0 |  |
| Yes | 0.80 (0.45, 1.43) | 0.4511 | 0.81 (0.45, 1.45) | 0.4767 |
| Peripheral Nerve Invasion |  |  |  |  |
| No | 1.0 |  | 1.0 |  |
| Yes | 1.33 (0.84, 2.12) | 0.2246 | 1.30 (0.81, 2.07) | 0.2740 |
| Lymphovascular Invasion |  |  |  |  |
| No | 1.0 |  | 1.0 |  |
| Yes | 1.27 (0.88, 1.82) | 0.2036 | 1.24 (0.86, 1.78) | 0.2569 |

Abbreviations: SII, systemic inflammation index; CIS, carcinoma in situ; OR, odds ratio; CI, confidence interval.

Univariate model adjust for: None
Multivariate model adjust for: Age; BMI; Gender; Smoking history; HBP; DM
